# The changing Amazon forest

**DOI:** 10.1098/rstb.2007.0033

**Published:** 2008-02-11

**Authors:** Oliver L Phillips, Simon L Lewis, Timothy R Baker, Kuo-Jung Chao, Niro Higuchi

**Affiliations:** 1 Earth and Biosphere Institute, School of Geography, University of LeedsLeeds LS2 9JT, UK; 2 Lab Silvicultura Tropical, Instituto Nacional de Pesquisas da AmazôniaManaus, Amazonas 69060-001, Brazil

**Keywords:** ecological change, growth, mortality, biomass, carbon sink

## Abstract

Long-term monitoring of distributed, multiple plots is the key to quantify macroecological patterns and changes. Here we examine the evidence for concerted changes in the structure, dynamics and composition of old-growth Amazonian forests in the late twentieth century. In the 1980s and 1990s, mature forests gained biomass and underwent accelerated growth and dynamics, all consistent with a widespread, long-acting stimulation of growth. Because growth on average exceeded mortality, intact Amazonian forests have been a carbon sink. In the late twentieth century, biomass of trees of more than 10 cm diameter increased by 0.62±0.23 t C ha^−1^ yr^−1^ averaged across the basin. This implies a carbon sink in Neotropical old-growth forest of at least 0.49±0.18 Pg C yr^−1^. If other biomass and necromass components are also increased proportionally, then the old-growth forest sink here has been 0.79±0.29 Pg C yr^−1^, even before allowing for any gains in soil carbon stocks. This is approximately equal to the carbon emissions to the atmosphere by Amazon deforestation. There is also evidence for recent changes in Amazon biodiversity. In the future, the growth response of remaining old-growth mature Amazon forests will saturate, and these ecosystems may switch from sink to source driven by higher respiration (temperature), higher mortality (as outputs equilibrate to the growth inputs and periodic drought) or compositional change (disturbances). Any switch from carbon sink to source would have profound implications for global climate, biodiversity and human welfare, while the documented acceleration of tree growth and mortality may already be affecting the interactions among millions of species.

## 1. Introduction

Given the scale of the human experiment with the biosphere it is evident that our activities now affect all Earth's ecosystems (Crutzen 2002). Processes such as deforestation are obvious; others, such as hunting and surface fires, are subtler but affect biodiversity in insidious ways (cf. Lewis *et al*. 2004*a*; Malhi & Phillips 2004). Atmospheric change will become increasingly significant, as carbon dioxide concentrations will reach levels unprecedented for at least 20 Myr (e.g. Retallack 2001) and climates move beyond Quaternary limits (Meehl *et al*. 2007). Moreover, the rate of change in these basic ecological drivers and constraints is without precedent in the evolutionary span of most species on Earth.

Biodiversity change as a consequence of recent climate change is widely documented in better-studied temperate areas (e.g. Parmesan & Yohe 2003), but tropical monitoring has been largely piecemeal and localized. Since 2000, we and others have developed a standardized, international, long-term network of permanent plots in mature forests across Amazonia, which unites existing efforts of local botanists and foresters, often working hitherto largely in isolation. This network of Amazon-forest researchers, known as ‘RAINFOR’ (*Red Amazónica de Inventarios Forestales*, or Amazon Forest-Inventory Network, http://www.geog.leeds.ac.uk/projects/rainfor/, Malhi *et al*. 2002), represents the combined long-term ecological monitoring efforts of 35 institutions worldwide. As well as using standard methods, RAINFOR participants share a desire to combine multiple, local efforts to help reveal the larger-scale patterns and processes which single site studies cannot. Here we synthesize recent results from the network to assess how old-growth Amazonian forests changed in the late 20th century in terms of forest structure, dynamics and composition.

## 2. Material and methods

Repeated long-term measurements of trees allow calculation of (i) the cross-sectional area that tree trunks occupy (‘basal area’), which can be used with allometric equations to estimate biomass, (ii) tree growth, (iii) the total number of stems present, (iv) stem recruitment (number of stems added to a plot over time), and (v) mortality (either the number or basal area of stems lost from a plot over time). We present results from 50 to 91 plots, depending upon selection criteria for different analyses, as described in Phillips *et al*. (in press). Further methodological details are given elsewhere (e.g. Baker *et al*. 2004*a*).

The plots span Amazonia (figure 1), including Bolivia, Brazil, Ecuador, French Guiana, Peru and Venezuela, from the driest southeast to the wettest northwest and the least fertile east to the most fertile west. Most are 1 ha in size, comprising approximately 600 trees of 10 cm diameter and above (at 1.3 m or above stem irregularities, following convention). Many have been monitored for more than a decade, starting as early as 1971. Here we synthesize findings across the network for censuses completed up to *ca* 2000—these data have all been published before but not in one synthetic article. In addition, we present a new plot-based estimate of the carbon balance of old-growth tropical forests in the late twentieth century. Details of plot locations and methods are explained elsewhere (Malhi *et al*. 2002; Phillips *et al*. 2002*a*,*b*, 2004; Baker *et al*. 2004*a*,*b*; Lewis *et al*. 2004*b*). Scaling from individual tree to biomass is based on diameter-based allometric equations (Baker *et al*. 2004*a*). We summarize findings from old-growth forests in terms of (i) structural changes, (ii) dynamic-process changes, and (iii) compositional changes, over the past two to three decades. We scale-up structural changes for the entire tropical forests based on forest cover (Mayaux *et al*. 2005) and allometric expansion factors.

## 3. Results

### (a) Structural changes

In old-growth Amazonia, above-ground biomass increased significantly between the first measurement (late twentieth century, mean date 1988) and the last measurement (mean date 2000). For trees of more than 10 cm diameter, the increase was 0.62±0.23 t C ha^−1^ yr^−1^ (mean±95% CI; Baker *et al*. 2004*a*). The above-ground biomass change is normally distributed (figure 2) and has occurred across regions and environmental gradients and through time (e.g. Baker *et al*. (2004*a*) for site-by-site data and Lewis *et al*. (2004*b*) for interval-by-interval results). The estimated increase is statistically indistinguishable from earlier estimates (Phillips *et al*. 1998) of 0.54±0.29 t C ha^−1^ yr^−1^ for the lowland Neotropics and 0.49±0.29 t C ha^−1^ yr^−1^ for Amazonia, both up to 1996, indicating continued biomass sink strength through to the end of the century.

We adopt a simple approach to scale-up plot-based biomass change estimates to larger areas (table 1). Thus, we assume that our measurements are an unbiased sample of the forest landscape; other biomass and necromass components also increased proportionally; and soil carbon stocks have been static, and estimate the magnitude of the South American carbon sink by multiplying the plot-based rate by a series of expansion factors and a mid-range estimate of the South American forest area in 2000 (7.8 Mkm^2^, Mayaux *et al*. 2005). This yields a total estimated late twentieth-century continental forest sink of 0.79±0.29 Pg C yr^−1^. If tropical forests elsewhere behaved similarly, then the combined old-growth tropical forest sink would be 1.60±0.58 Pg C yr^−1^, before accounting for any change in soil carbon stock. We present a range of estimates in table 1, broken down by biomass component, forest area estimate and continent. The precise values depend on various assumptions, but they all imply a substantial carbon flux to mature forests. The magnitude is similar to the land–atmosphere flux caused by tropical deforestation. Updated results from Amazonia and ground-based studies from other major tropical forests are urgently required, but Amazonia, at least, was approximately at carbon balance over the final years of the twentieth century in spite of deforestation emissions. This is consistent with the evidence from recent global inversions of atmospheric CO_2_ measurements and local aircraft measurements of atmospheric CO_2_ profiles, showing that the tropics are either carbon-neutral or sink regions, despite widespread deforestation (Denman *et al*. 2007, p. 522; Stephens *et al*. 2007).

### (b) Dynamic changes

Amazon forests not simply gained mass, they became more dynamic too. We measured the dynamics of forests in two ways. Firstly, we examined changes in stem population dynamics. We estimated stem turnover between censuses as the mean of annual mortality and recruitment rates for the population of trees of 10 cm diameter and above. Secondly, we examined the changes in basal area fluxes of the forest—in terms of basal area growth gains and mortality losses.

Among all 50 old-growth plots across tropical South America with at least three censuses (i.e. at least two consecutive monitoring periods), six key ecosystem processes—stem recruitment, mortality and turnover, and basal area growth, loss and turnover—increased significantly (figure 3) between the first and second monitoring periods (Lewis *et al*. 2004*b*). Thus these forests became on average faster growing and more dynamic. Proportionally, the annual increases in the dynamic fluxes (growth, recruitment and mortality: 1–3%) were almost an order of magnitude greater than the increases in the structural pools (basal area and stem density: 0.1–0.4%). Increases are not the short-term results of weather fluctuations: average recruitment rates consistently exceeded mortality rates, and mortality lagged recruitment (figure 4). Moreover, the increases occurred across both east and west Amazonia (Lewis *et al*. 2004*b*; Phillips *et al*. 2004). Although the greatest *absolute* increases in rates were in the more dynamic sites of the west, the *proportional* increases in rates have been equivalent among forest types (Lewis *et al*. 2004*b*). Increasing growth, recruitment and mortality thus occurred simultaneously and across different geographical environments over the late twentieth century.

Changes in the structure and dynamics of tropical forests are likely to be accompanied by changes in species composition and forest function. We studied woody climbers (structural parasites on trees, also called lianas) that contribute to approximately 20% of forest leaf productivity: across western Amazonia the density and dominance of large lianas increased over the last two decades of the twentieth century (Phillips *et al*. 2002*b*). In addition, a large cluster of plots in central Amazonia shows changes in tree species composition over the same period (Laurance *et al*. 2004). There have been pervasive changes here: growth, mortality and recruitment all increased significantly over two decades (basal area also increased, but not significantly so), with faster-growing genera showing larger absolute and relative increases in growth, relative to slower-growing genera. Further studies are urgently required to determine whether comparable shifts in tree communities are occurring throughout Amazonia.

## 4. Discussion

### (a) What has been driving these changes?

In sum, there have been simultaneous changes in tree growth, recruitment, mortality, stem density, biomass and (probably) composition in the late twentieth-century forests across tropical South America. Most debate has centred on the relatively mundane finding of increased biomass (e.g. Clark 2002; Wright 2005) and not on the larger picture of changed (accelerated) forest dynamics. We lack the space to review these debates in detail (further discussion is provided in Phillips *et al*. 2002*a*,*b*, 2004; Baker *et al*. 2004*a*; Malhi & Phillips 2004). Our contention is that the spatial, environmental and temporal coherence of the pattern (east and west, wet and dry, rich and poor soil, before, through, and after the major 1997–1998 El Niño event), and its coincidence with growth and mortality increases show that Amazonian old-growth forests were at general disequilibrium. In particular, the lack of obvious impacts of drought, the simultaneous long-term increases in growth (Lewis *et al*. 2004*a*,*b*) and stem density (Phillips *et al*. 2004) within and among plots, and the lack of large unexplained necromass stocks in our plots (Baker *et al*. 2007; Chao 2007) contradict the notion that the biomass increases observed simply result from recovery from earlier unobserved disturbance(s).

The simplest explanation for the ensemble result—more biomass, more stems, faster recruitment, faster mortality, faster growth and more lianas—is that improved resource availability has increased net primary productivity, in turn increasing growth rates (Lewis *et al*. 2004*a*,*b*). This would account for the increase in stand basal-area growth and stem recruitment rates, and the fact that these show the most highly significant changes and predate the mortality increases (Lewis *et al*. 2004*b*). Owing to increased growth, competition for limiting resources, such as light, water and nutrients, increases. Over time, some of the faster growing, larger trees die, as do some of the ‘extra’ recruits (the accelerated growth percolates through the system). This accounts for the increased losses from the system, shown by increase in mortality rates. Thus, the system gains biomass and stems, while the losses lag some years behind, causing an increase in above-ground biomass and stems. Overall, this suite of changes is explicable qualitatively by a long-term increase in a limiting resource.

Increasing resource availability could also explain the compositional changes. Lianas may respond either to rising resource supply rates or to greater disturbance caused by higher tree-mortality rates, or both. Changing tree composition in central Amazonia is also consistent with increasing resource supply rates, as experiments show that faster-growing species are often the most responsive in absolute terms to increases in resource levels (Coomes & Grubb 2000), although some argue (e.g. Körner 2004; J. Lloyd 2007, personal communication) that the greatest proportional response should be in understorey seedlings and saplings which are likely to be close to carbon deficit due to shading—a small increase in photosynthetic rate here having a great proportional impact on carbon balance. There is indeed some experimental evidence to support this view (e.g. Kerstiens 2001; Aidar *et al*. 2002), but seedlings and saplings have not been monitored in the RAINFOR network to date.

What kind of environmental changes could have increased the growth and productivity of tropical forests? Elsewhere we have discussed the candidate drivers in detail (Lewis *et al*. 2004*a*; Malhi & Phillips 2004; Lewis 2006). While there have been widespread changes in the physical, chemical and biological environment of tropical trees, the only change for which there is unambiguous evidence that the driver has widely changed *and* that such a change should accelerate forest growth (Lewis *et al*. 2004*a*) is the increase in atmospheric CO_2_. The undisputed long-term increase in concentrations, the key role of CO_2_ in photosynthesis, and the demonstrated effects of CO_2_ fertilization on plant growth rates make this the primary candidate. However, a substantial role for increased insolation (e.g. Ichii *et al*. 2005) or aerosol-induced increased diffuse fraction of radiation (e.g. Oliveira *et al*. 2007) cannot be ruled out.

### (b) The future: susceptibility of Amazon forest to environmental stress and compositional changes

The world's largest remaining tract of tropical forest underwent concerted changes in forest dynamics over the late twentieth century. Such rapid change was not anticipated, suggesting that other surprises might arise as global changes accelerate in coming decades. Tropical forests are evidently sensitive to atmospheric changes.

Old-growth Amazonian forests have also evidently helped to slow the rate of CO_2_ accumulation in the atmosphere, thereby slowing down global climate change. The concentration of atmospheric CO_2_ is rising at an annual rate equivalent to approximately 4 Pg C; this could be 13–28% greater without the annual Neotropical biomass carbon sink of 0.5–1.1 Pg C, and 25–50% greater if other tropical forests are behaving in a similar way. This subsidy from nature may be a relatively short-lived phenomenon. Mature Amazonian forests will either (i) continue to be a *carbon sink* for decades (Chambers *et al*. 2001; Cramer *et al*. 2001) or (ii) soon become *neutral or a small carbon source* (Cramer *et al*. 2001; Phillips *et al*. 2002*b*; Körner 2004; Laurance *et al*. 2004) or (iii) become a *mega-carbon sourc*e (Cox *et al*. 2000; Lewis 2006). Given that a 0.4% annual increase in Amazon forest biomass roughly compensates for the entire fossil fuel emissions of Western Europe (or the deforestation in Amazonia), a switch from a moderate carbon sink to even neutral or a moderate carbon source would have implications for global climate and human welfare. Approximately, 0.4% of annual sink represents the difference between two much larger values: stand-level growth (averaging approx. 2%) and mortality (averaging approx. 1.6%), so *either* a small decrease in growth *or* a small increase in mortality could shut the sink down. Logically, mortality will inevitably catch up with growth at some point as the system tends to a new, quasi-equilibrium state at higher biomass. There are several further mechanisms by which such a switch could occur, apart from the obvious and immediate threats posed by land use change and associated disturbances by fragmentation and fire.

#### (i) Temperature and CO_2_ effects

Intact forests will remain a sink as long as carbon uptake associated with photosynthesis exceeds the carbon efflux from respiration. Under the simplest scenario of a steady rise in forest productivity over time, forests could remain a sink for decades (e.g. Lloyd & Farquhar 1996; Chambers *et al*. 2001). However, the current increases in productivity, apparently caused by continuously improving conditions for tree growth, cannot continue indefinitely. If carbon dioxide is the cause then trees are likely to become CO_2_ saturated (i.e. limited by another resource) at some point in the future. More generally, whatever the driver for recently accelerated growth, other factors such as soil nutrients will eventually limit productivity.

Rising temperatures could also shrink the forest sink. Warmer temperatures increase the rates of virtually all chemical and biological processes in plants and soils (including the enhancement of any CO_2_ fertilization effect), until temperatures reach inflection points where enzymes and membranes lose functionality. Canopy-to-air vapour deficits and stomatal feedback effects may be paramount in how tropical forest photosynthesis responds to future climate change (Lloyd *et al*. 1996).

The relationship between temperature changes and respiration is also critical. The most severe modelled outcomes for Amazonia (large-scale dieback) depend not only on drought-induced mortality but also on elevated soil respiration under increased temperatures (e.g. Cox *et al*. 2000). Some carbon loss from respiration will almost certainly increase as air temperatures continue to increase. Meanwhile, carbon gains from photosynthesis cannot rise indefinitely and will almost certainly asymptote. Thus, ecophysiological principles alone suggest that the sink in intact tropical forests will diminish and may eventually reverse. The major uncertainty is *when* this will occur.

#### (ii) Moisture and radiation

Climate change includes alterations to regional and global precipitation patterns. There are critical thresholds of water availability below which closed tropical forest does not persist and is replaced by savannah; the current threshold of 1300–1500 mm rainfall per annum (Salzmann & Hoelzmann 2005) could increase with rising temperatures. Thus, increasing temperatures and/or changing precipitation patterns may cause shifts in vegetation from carbon-rich tropical forests to ‘carbon-light’ savannah systems. Whether Amazon forests are ecophysiologically resilient to droughts is a subject of active research, reviewed elsewhere (Lloyd & Farquhar 2008).

What is the evidence so far of drought impacting Amazon forests? The temporal resolution of RAINFOR plots has generally been insufficient to attribute growth and mortality to individual years. Nevertheless, among the longest running plots (initiated in the 1970s or earlier), the severe 1982–1983 El Niño event did not greatly affect forest dynamics (Phillips 1995). Where there are annual or higher-resolution records, there is some evidence of short-term stand-level rates responding to moisture stress, with growth decreasing markedly in the dry season near Rio Branco, Acre (Vieira *et al*. 2004) and mortality temporarily increasing during the 1997–1998 El Niño near Manaus (Williamson *et al*. 2000), but the effects of that drought were negligible in relation to the long-term sink when averaged across all RAINFOR sites. Indeed, the impact on growth rates of moderate dry conditions in Amazonia may not necessarily be negative. There is evidence from leaf level (Graham *et al*. 2003) and at regional scales (Myneni *et al*. 2007) to suggest that Neotropical moist forests may be as strongly light-limited as they are moisture-limited. If so, while droughts reduce productivity and exacerbate fire risk in more marginal forest locations, the pan-Amazon impact on growth of more cloud-free rainless days could still be positive. Net impacts will depend exactly on when and where any drying is concentrated. The recent strong drought of 2005 will provide a direct test of the potential of drought to impact the long-term carbon sink.

#### (iii) Compositional change

Biodiversity change has consequences for climate change because plant species vary in the rate at which they store and process carbon. Yet, most models that project the future carbon balance in Amazonia (and future climate change scenarios) make no allowance for changing forest composition. One plausible outcome is a shift to faster growing, more light-demanding species, driven by increasing frequency of mortality, gap formation and liana infestation (Phillips & Gentry 1994; Körner 2004; Phillips *et al*. 2004, 2005). Fast-growing species generally have lower wood density and hence less carbon than slow-growing trees. The scope for biodiversity change impact on carbon storage is highlighted by simulation studies of Bunker *et al*. (2005): if slower-growing tree taxa are lost from an accelerated forest then up to one-third of the biomass carbon storage capacity would disappear. In Amazonia, there is currently an approximately 20% difference in mean wood density of the ‘faster’ forests in the west compared with slower forests in the east. Because faster forests also have lower basal area, differences in terms of carbon stored are greater still (figure 5).

## 5. Conclusions

In sum: (i) surviving tropical forests have already changed substantially (more biomass, more stems, faster recruitment, faster mortality, faster growth and more lianas), (ii) these changes had global impacts in terms of carbon sequestration, (iii) the simplest explanation of these results invokes a multidecadal and continental or global scale growth stimulation—all observed results follow self-evidently from this, and (iv) further changes are inevitable given the number of ecological drivers that have been perturbed. Over the coming decades the biomass carbon sink will certainly diminish, due to increasing resource limitation and respiration, and drought presents an additional threat. Compositional change driven by greater resource supply and selection for faster-growing trees may also help shut down or reverse the carbon sink function of forests. Together with the direct human impacts on Amazonia, these ecological and biogeochemical changes are significant threats to global climate, biodiversity and human well-being. Maintaining a fully standardized, international network of long-term plots extending across many dozens of localities will be essential if we are to monitor and test for the impacts of such changes and provide some early warning.

## Figures and Tables

**Figure 1:**
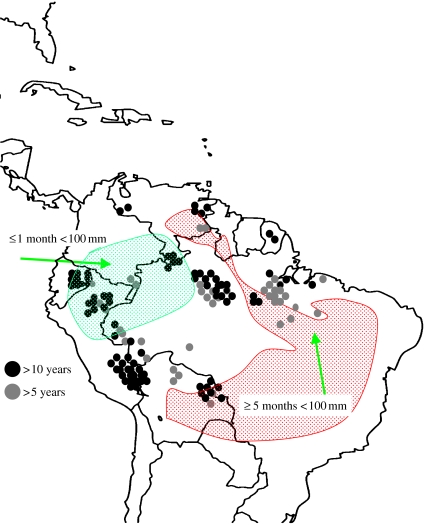
Plot locations. Symbols represent approximate locations of each plot: grey circles for plots monitored for 5–10 years and black circles for those with more than 10 years of monitoring. The approximate extent of less and more seasonal areas of tropical South America is indicated.

**Figure 2:**
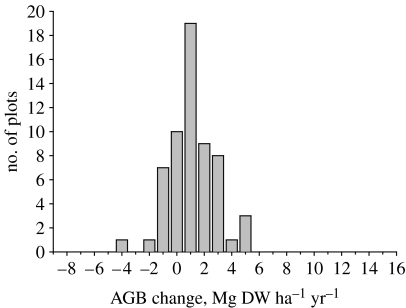
Above-ground biomass (AGB) change (dry weight; DW) of trees of 10 cm diameter and above in 59 Amazon plots, based on initial and final stand-biomass estimates (Baker *et al*. 2004*a*). As expected, for a random sample of small plots measured for a finite period, some sites show a decline in biomass during that period indicating mortality that exceeded tree growth at that point in space and time. Both mean and median are shifted to the right of zero (*p*<0.01).

**Figure 3:**
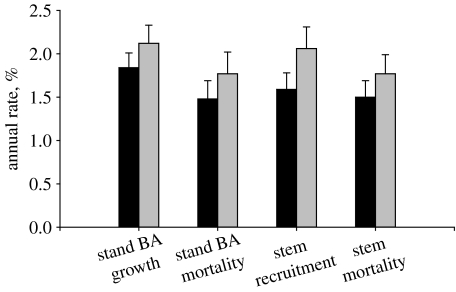
Annualized stand level basal-area (BA) growth, basal-area mortality, stem recruitment and stem mortality rates from all 50 plots with two consecutive census intervals (Lewis *et al*. 2004*b*); mean with 95% CIs. All increases are significant (paired *t*-tests). The average mid-year of the first (black bars) and second (grey bars) intervals was 1989 and 1996, respectively.

**Figure 4:**
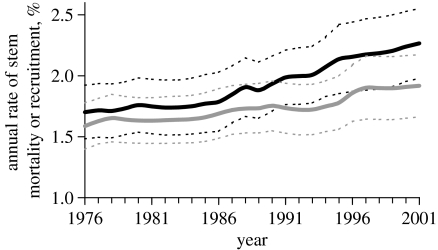
Mean and 95% CIs for stem recruitment and mortality rates against calendar year, for plots across Amazonia. Black lines indicate recruitment and grey lines indicate mortality; solid lines are means and dotted lines are 95% CIs. Rates were corrected for the effects of differing census-interval lengths, for site-switching and possible ‘majestic-forest bias’ (Phillips *et al*. 2004). All trends hold if these corrections are not applied.

**Figure 5:**
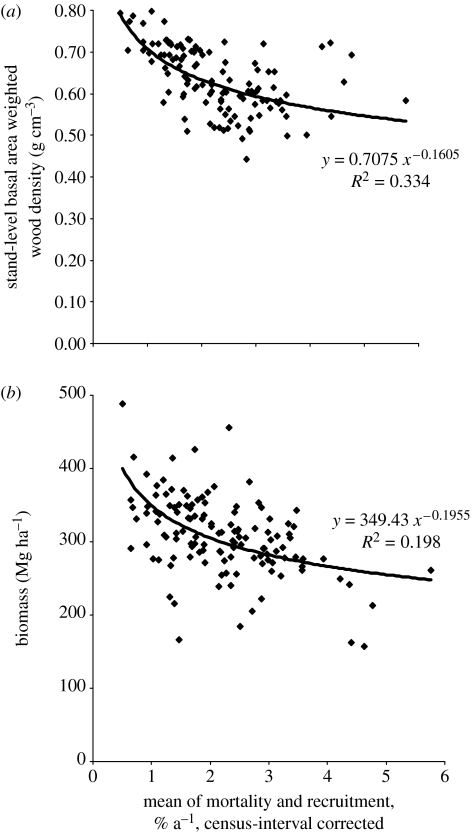
Biomass as a function of mean stand-level wood density for 127 lowland forest plots across South America; (*a*) initial stand level and (*b*) initial biomass as a function of subsequent annual stem turnover rate. Note that faster forests have lower wood density and much lower biomass, in spite of substantial variation attributable to other factors.

**Table 1:** Estimated late twentieth-century net carbon sink in different components of biomass and different geographical regions, across the world's major tropical forests. (We take the net gain in above-ground coarse biomass (trees of 10 cm dbh and above) recorded in Amazonia (0.62±0.22 t C ha^−1^ yr^−1^), and scale by the estimated ratio of trees of up to 10 cm dbh and lianas of 1 cm dbh and above to trees of 10 cm dbh and above in Amazonia (equal to 0.099, Phillips *et al*. 1998), by the most comprehensive estimate of coarse necromass:above-ground coarse biomass ratio available for Amazonia (equal to 0.127, K.-J. Chao, O. L. Phillips, T. R. Baker 2002–2006, unpublished data), and by the latest estimate of below-ground:above-ground biomass ratio (equal to 0.370, unpublished central Amazonian estimate: N. Higuchi 2000–2006, unpublished data). The values for each region are estimated by assuming the same allometry and behaviour as Amazonian forests. Forest area estimates are adapted from Mayaux *et al*. (2005). GLC, Global Land Cover; FRA CS, FAO Forest Resource Assessment (2000) country statistics; FRA RS, FAO Forest Resource Assessment (2000) remotely sensed values. Scaled-up estimates based on FRA RS highlighted in italics are mentioned in the text. Units for biomass stock increases are 10^6^ t C yr^−1^.)

	land cover class	forest area	coarse above-ground biomass increase (trees of 10 cm dbh and above)		above-ground biomass increase (trees of up to 10 cm and lianas of 1 cm and above)	coarse necromass increase	total above-ground biomass and necromass increase		below-ground biomass increase	total biomass and necromass increase	
							
		ha 10^6^	mean	CI	mean	mean	mean	CI	mean	mean	CI
South America											
GLC2000	humid tropical forest	630.5	392.2	142.4	38.8	49.8	480.9	174.6	159.4	640.3	232.4
	dry tropical forest	146.7	91.3	33.1	9.0	11.6	111.9	40.6	37.1	149.0	54.1
	flooded tropical forests	25.3	15.7	5.7	1.6	2.0	19.3	7.0	6.4	25.7	9.3
	total	802.5	499.2	181.2	49.4	63.4	612.1	222.2	202.9	815.0	295.8
FRA CS	closed forest	858.3	533.9	193.8	52.9	67.8	654.6	237.6	217.0	871.7	316.4
	open forest	68.9	42.9	15.6	4.2	5.4	52.5	19.1	17.4	70.0	25.4
	total	927.2	576.8	209.4	57.1	73.3	707.2	256.7	234.5	941.6	341.8
FRA RS	forest total	780.2	*485.4*	*176.2*	48.1	61.6	595.1	216.0	197.3	*792.3*	*287.6*
Africa											
GLC2000	humid tropical forest	232.7	144.8	52.5	14.3	18.4	177.5	64.4	58.8	236.3	85.8
	dry tropical forest	415.1	258.2	93.7	25.6	32.8	316.6	114.9	105.0	421.6	153.0
	flooded tropical forests	13.1	8.1	3.0	0.8	1.0	10.0	3.6	3.3	13.3	4.8
	total	660.9	411.1	149.2	40.7	52.2	504.1	183.0	167.1	671.2	243.6
FRA CS	closed forest	352.7	219.4	79.6	21.7	27.9	269.0	97.6	89.2	358.2	130.0
	open forest	288.9	179.7	65.2	17.8	22.8	220.3	80.0	73.1	293.4	106.5
	total	641.6	399.1	144.9	39.5	50.7	489.3	177.6	162.2	651.6	236.5
FRA RS	forest total	518.5	322.6	117.1	31.9	41.0	395.5	143.6	131.1	*526.6*	*191.1*
Asia											
GLC2000	humid tropical forest	230.6	143.5	52.1	14.2	18.2	175.9	63.8	58.3	234.2	85.0
	dry tropical forest	144.8	90.1	32.7	8.9	11.4	110.4	40.1	36.6	147.1	53.4
	flooded tropical forests	13.5	8.4	3.0	0.8	1.1	10.3	3.7	3.4	13.7	5.0
	total	388.9	241.9	87.8	24.0	30.7	296.6	107.7	98.3	395.0	143.4
FRA CS	closed forest	416.2	258.9	94.0	25.6	32.9	317.4	115.2	105.2	422.7	153.4
	open forest	58.3	36.3	13.2	3.6	4.6	44.5	16.1	14.7	59.2	21.5
	total	474.5	295.2	107.2	29.2	37.5	361.9	131.4	120.0	481.9	174.9
FRA RS	forest total	272.0	169.2	61.4	16.8	21.5	207.5	75.3	68.8	*276.2*	*100.3*
global											
GLC2000	humid tropical forest	1093.8	680.5	247.0	67.4	86.4	834.2	302.8	276.6	1110.8	403.2
	dry tropical forest	706.6	439.6	159.6	43.5	55.8	538.9	195.6	178.7	717.6	260.5
	flooded tropical forests	51.9	32.3	11.7	3.2	4.1	39.6	14.4	13.1	52.7	19.1
	total	1852.3	1152.3	418.3	114.1	146.3	1412.7	512.8	468.4	1881.1	682.9
FRA CS	closed forest	1627.2	1012.3	367.5	100.2	128.6	1241.1	450.5	411.5	1652.5	599.9
	open forest	416.1	258.9	94.0	25.6	32.9	317.4	115.2	105.2	422.6	153.4
	total	2043.3	1271.1	461.4	125.8	161.4	1558.4	565.7	516.7	2075.1	753.3
FRA RS	forest total	1570.7	977.1	354.7	96.7	124.1	1198.0	434.9	397.2	*1595.1*	*579.1*
